# Hormonal Contraceptives Differentially Suppress TFV and TAF Inhibition of HIV Infection and TFV-DP in Blood and Genital Tract CD4+ T cells

**DOI:** 10.1038/s41598-017-18078-3

**Published:** 2017-12-18

**Authors:** Zheng Shen, Marta Rodriguez-Garcia, Mickey V. Patel, Jack Bodwell, Angela D. M. Kashuba, Charles R. Wira

**Affiliations:** 1 Department of Microbiology and Immunology, Geisel School of Medicine at Dartmouth, Lebanon, NH USA; 2 Division of Pharmacotherapy and Experimental Therapeutics, University of North Carolina Eshelman School of Pharmacy, Chapel Hill, NC USA

## Abstract

HIV prevention research is focused on combining antiretrovirals (ARV) and progestin contraceptives to prevent HIV infection and pregnancy. The possibility that progestins compromise ARV anti-HIV activity prompted us to evaluate the effects of progestins on tenofovir (TFV) and TFV-alafenamide (TAF) on HIV infection and intracellular TFV-diphosphate (TFV-DP) concentrations in blood and genital CD4+ T cells. Following incubation of blood CD4+ T cells with TFV or TAF, Medroxyprogesterone acetate (MPA), but not Levonorgestrel, Norethisterone or progesterone, suppressed the anti-HIV effect of TFV by reducing intracellular TFV-DP, but had no effect on TAF inhibition of infection or TFV-DP. In contrast, with genital CD4+ T cells, MPA suppressed TAF inhibition of HIV infection and lowered of TFV-DP concentrations without affecting TFV protection. These findings demonstrate that MPA selectively compromises TFV and TAF protection in blood and genital CD4+ T cells and suggests that MPA may decrease ARV protection in individuals who use ARV intermittently for prevention.

## Introduction

Worldwide, women represent half of the 36.7 million people living with Human Immunodeficiency Virus (HIV)^1^. In Sub-Saharan Africa, women comprise 59% of infected individuals, with young women (age 15–24) accounting for 22% of all new infections. A young woman is infected every minute and HIV infection represents the main cause of death in women of reproductive age^2^.

Sexual transmission of HIV is the main route for HIV acquisition in women^3^. Therefore, to prevent HIV acquisition, protection against HIV infection needs to be achieved in the female reproductive tract (FRT), the more common portal of HIV entry for women. One strategy for HIV prevention in women is systemic or local administration of ARVs for pre-exposure prophylaxis (PrEP). Currently, Truvada (tenofovir disoproxil fumarate (TDF) in combination with emtricitabine) is approved for oral PrEP^4^, after showing efficacy in several clinical trials including those with heterosexual couples^5,6^. However, trials designed to assess the effectiveness of Truvada or TDF in preventing HIV infection specifically in women were unable to demonstrate protection after oral administration^7,8^. Vaginal application of tenofovir (TFV) has also been tested as a PrEP strategy in different trials, with moderate success in one out of three studies^7,9,10^. While lack of compliance was a major contributor to the failure of these trials^11^, similar adherence rates (measured as detectable plasma concentrations) in male and female only trials (iPrEX and VOICE) resulted in very different protection rates (44 and −4% respectively)^7,12^. These results suggest that biological factors unique to women may also be involved. For example, analysis of intracellular TFV diphosphate (TFV-DP; the active form of TFV responsible for anti-HIV activity) after oral administration of a single dose of TDF demonstrated that concentrations were a 100-fold higher in rectal tissues when compared to vaginal and cervical tissues^13^. In other studies, measuring mucosal tissue concentrations of tenofovir following oral dosing, a minimum adherence to 6 of 7 doses/week (85%) was required to protect lower female genital tract tissues from HIV, while adherence to 2 of 7 doses/week (28%) was required to protect colorectal tissue^14^.

We have previously reported that female sex hormones have the potential for modifying TFV conversion to TFV-DP^15^. Also of relevance is the recent study that bacterial vaginosis-associated bacteria can affect TFV levels and HIV transmission risk^16^. While topical ARV administration offers advantages of higher vaginal drug concentrations and lower systemic toxicity, it is unclear whether protective concentrations of ARVs can be reached in the entire FRT after local ARV administration^17–19^. Protection of the entire FRT along with regional lymph nodes is necessary, given the evidence that HIV-target cells are present throughout the FRT^15,20–23^ and studies in non-human primate models showing that infection can occur in all the major anatomical regions of the FRT^21,22^.

A new pro-drug of TFV, Tenofovir alafenamide (TAF; formerly known as GS-7340), has been approved for HIV treatment^24^. TAF presents advantages compared to TFV, including increased anti-HIV efficacy, reduced toxicity, and preferential accumulation in lymphoid tissues and HIV susceptible cells^24–26^. Under clinical conditions, TAF is administered at lower doses than tenofovir (TFV), owing to the more efficient intracellular conversion of TAF into TFV-DP than TFV^24–26^. Moreover, TAF is a prodrug formulation of TFV that exerts antiviral activity at up to 300-fold lower levels. While TFV tissue concentrations after vaginal administration of TFV have been investigated, tissue concentrations of TAF are still under investigation and no studies using topical administration of TAF are available. TAF conversion to TFV inside the cells is initiated by the esterase activity of Cathepsin A, followed by subsequent reactions probably occurring within lysosomes to form TFV^27^, after which the same kinases are utilized to form TFV-DP. Despite the advantages that TAF offers for HIV treatment, it is not currently approved for Pre-Exposure Prophylaxis (PrEP)^28^. However, its ability to protect against mucosal HIV infection and determination of genital tissue concentrations are being investigated^20,29,30^.

In addition to HIV, a major health issue for women of reproductive age is unintended pregnancy. To meet the needs of reproductive age women, current HIV prevention research is leading towards multipurpose prevention technologies (MPTs). MPTs deliver hormonal contraceptives and ARVs to simultaneously prevent unintended pregnancy and HIV infection^31,32^. However, several studies have reported contradictory findings about the interactions between ARVs and hormonal contraceptives^33,34^. These findings emphasize the need to determine whether hormonal contraceptives interact with ARVs to decrease the effectiveness of ARVs in protecting against HIV.

In this study we evaluated possible interactions between progestin hormonal contraceptives (MPA, norethindrone (NET) and levonorgestrel (LNG)) and ARVs (TFV and TAF) that could result in decreased anti-HIV activity of ARVs. We compared interactions using CD4+ T cells from blood and from FRT tissues. Our experimental approach was to use lower concentrations of TFV and TAF that resulted in approximately 50% inhibition of HIV infection, and concentrations approximately 1500-fold higher to measure intracellular TFD-DP. We found that MPA, but not LNG or NET, was able to reverse the protective effects of ARVs. Unexpectedly, we found that MPA decreased TFV anti-HIV activity in blood CD4+ T cells and TAF anti-HIV activity in tissue CD4+ T cells. Increased infection after MPA treatment was associated with decreased intracellular TFV-DP, the active drug form with anti-HIV activity. These findings highlight the need to test potential hormonal contraceptive and ARV (topical and oral) interactions in blood and tissue cells when developing MPTs, since anti-HIV effectiveness may be differentially compromised by contraceptives based upon anatomical location.

## Results

### Intracellular TFV-DP concentrations in CD4+ T cells from Blood and FRT tissues

**Figure 1:**
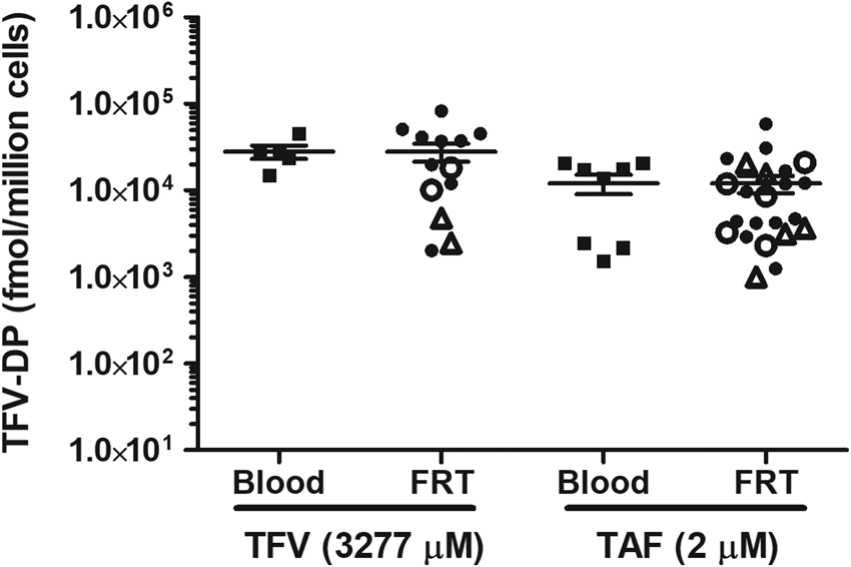
Intracellular TFV-DP concentrations from TFV/TAF treatment in CD4+ T cells from blood and female reproductive tract tissues. TFV-DP concentrations were measured by LC-MS/MS in purified CD4+ T cells from blood (n = 11) and from FRT (Endometrium: n = 17, Cervix: n = 5, Ectocervix: n = 5) treated with TFV (3277 µM) or TAF (2 µM) for 24hr. Values are expressed as fmol/million cells. Each symbol represents blood CD4+ T cells from different female donors or FRT CD4+ T cells from an individual patient. Dark squares indicate blood CD4+ T cells. Dark circles indicate endometrial CD4+ T cells. Open circles indicate cervix CD4+ T cells and triangles indicate ectocervix CD4+ T cells. The mean and SEM are shown.

The amount of TFV applied vaginally (40 mg total at a concentration of 10 mg/ml) in HIV prevention trials^9^ and the levels of TAF found in blood after oral administration^24–26^ are very different. Therefore, to accurately compare the effects of TFV and TAF, we predetermined the dose for each ARV which would give the same intracellular concentration of TFV-DP and therefore the same levels of protection against HIV infection. As seen in Fig. 1, blood CD4+ T cell incubation for 24hr with TFV (1 mg/ml: 3277 μM) or TAF (1 µg/ml: 2 μM) resulted in equivalent amounts of intracellular TFV-DP. Under identical culture conditions, incubation of CD4+ T cells from the FRT also resulted in intracellular concentrations of TFV-DP that were not significantly different from blood CD4+ T cells. Moreover, when CD4+ T cells from the endometrium (EM, black circle), endocervix (CX, open circle) and ectocervix (ECX, triangle) were analyzed, intracellular concentrations of TFV-DP between sites in the FRT were the same and not significantly different from the concentrations measured in blood CD4+ T cells. Each symbol represents blood CD4^+^ T cells from different female donors or FRT CD4+ T cells from an individual patient. These data indicate that irrespective of the source of CD4+ T cells analyzed, the doses of ARV selected result in intracellular concentrations of TFV-DP for blood and FRT CD4+ T cells that are approximately 1–2 × 10^4^ fmol/million cells. These data provide the foundation for studies in the following sections, which determine whether progestational contraceptives compromise both TFV and TAF mediated anti-HIV immune protection as well as intracellular concentrations of TFV-DP.

### Comparison of TFV and TAF Inhibition of HIV Infection of CD4+ T Cells from Blood and Endometrium

**Figure 2:**
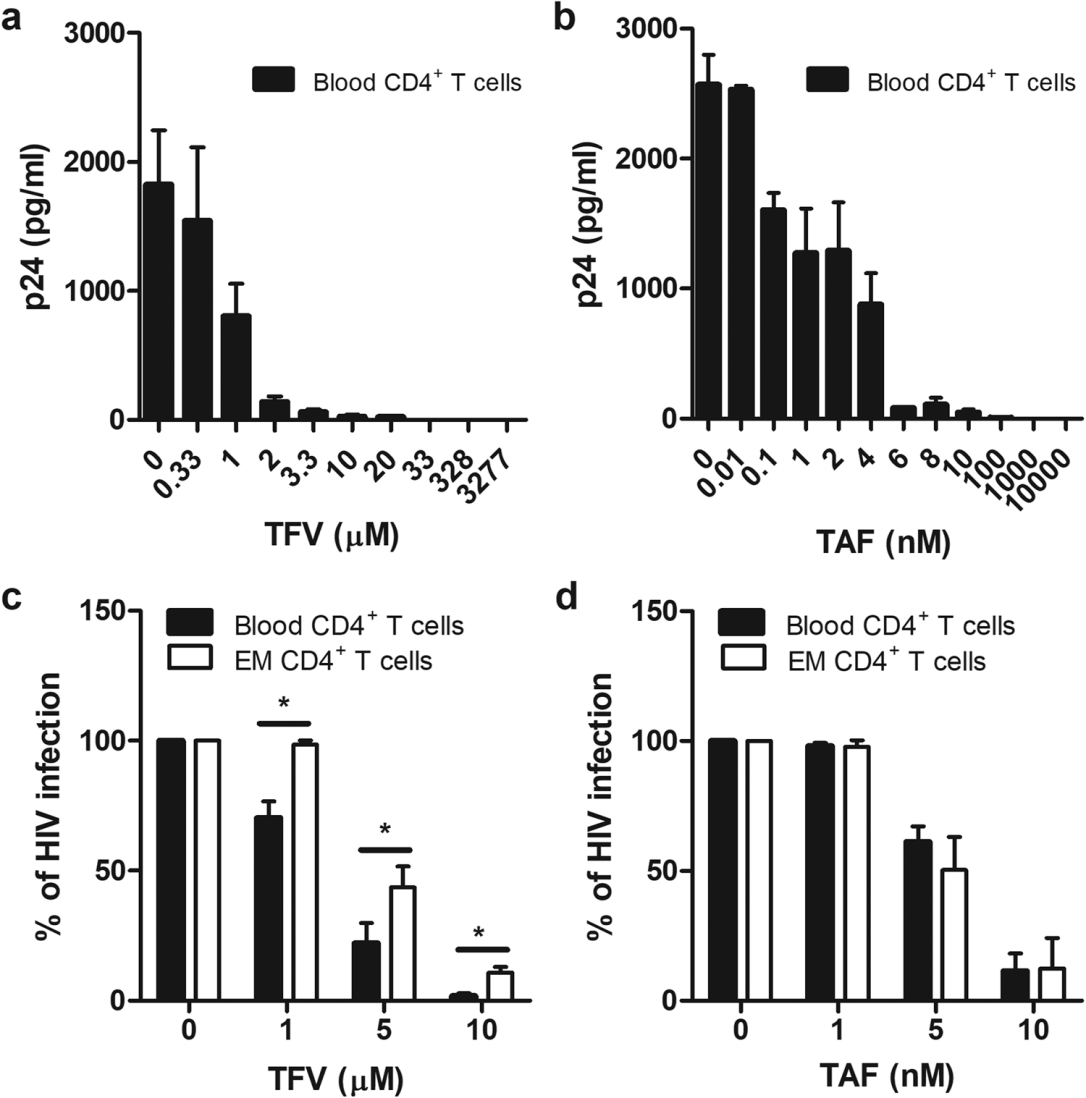
Dose response and comparison of TFV and TAF inhibition of HIV infection of CD4+ T cells from blood and endometrium (EM). Activated CD4+ T cells from blood and endometrium were pre-treated with TFV or TAF for 24hr and inoculated with HIV for 2hr. Released p24 levels in the culture media after 5 days of infection were measured by p24 enzyme-linked immunosorbent assay. Released p24 levels into the culture media after 5 days of infection when blood CD4+ T cells from same female donor were pre-treated with indicated doses of TFV (**a**) or TAF (**b**). The bar represents the mean and SEM from triplicate cultures. Effect of increasing concentrations of TFV (**c**) and TAF (**d**) on inhibition of HIV in CD4+ T cells from blood (n = 6) and endometrium (n = 3). Data were normalized to % of HIV infection with respect to infected control without TFV or TAF pre-treated. The bar represents the mean and SEM. *p < 0.05.

The doses of TFV and TAF used in Fig. 1 are very efficient at suppressing HIV infection of CD4+ T cells *in vitro*, and therefore could mask any potential modifications of ARV effectiveness by contraceptives. For this reason, dose response studies were undertaken to identify the concentration of TFV and TAF that resulted in approximately 50% inhibition of HIV infection, in order that changes in effectiveness in either direction (increase or decrease) could be detected. Following activation for 24hr, purified blood CD4+ T cells were treated with TFV (0.33–3277 μM) or TAF (0.01–10,000 nM) for another 24hr prior to extensive washout and infection with BaL (MOI 0.1) as described in Methods. In dose response studies, we found that inhibition of HIV infection by TFV (Fig. 2a) was lost between 1–10 μM, while TAF (Fig. 2b) inhibition of HIV infection was lost at doses between 1–10 nM.

To determine whether CD4+ T cells from the endometrium and blood are equally protected against HIV infection by TFV and TAF, CD4+ T cells were incubated with increasing doses of TFV and TAF for 24hr prior to infection with Bal (MOI 0.1). As seen in Fig. 2c, whereas blood CD4+ T cell infection was partially decreased by TFV starting at 1 μM, the same 1 μM dose of TFV had no anti-HIV effect on endometrial CD4+ T cells. The anti-HIV effect of TFV was also significantly reduced in endometrial CD4+ T cells at 5 and 10 μM when compared to blood CD4+ T cells. In contrast to TFV, as shown in Fig. 2d, TAF inhibited HIV infection to the same extent in blood and endometrial CD4+ T cells with no differences seen at the 3 concentrations tested. Overall, these findings indicate that endometrial CD4+ T cells are different from blood CD4+ T cells in terms of their sensitivity to TFV but not TAF.

### Effect of MPA on TFV inhibition of HIV Infection and intracellular TFV-DP in CD4+ T cells from blood

**Figure 3:**
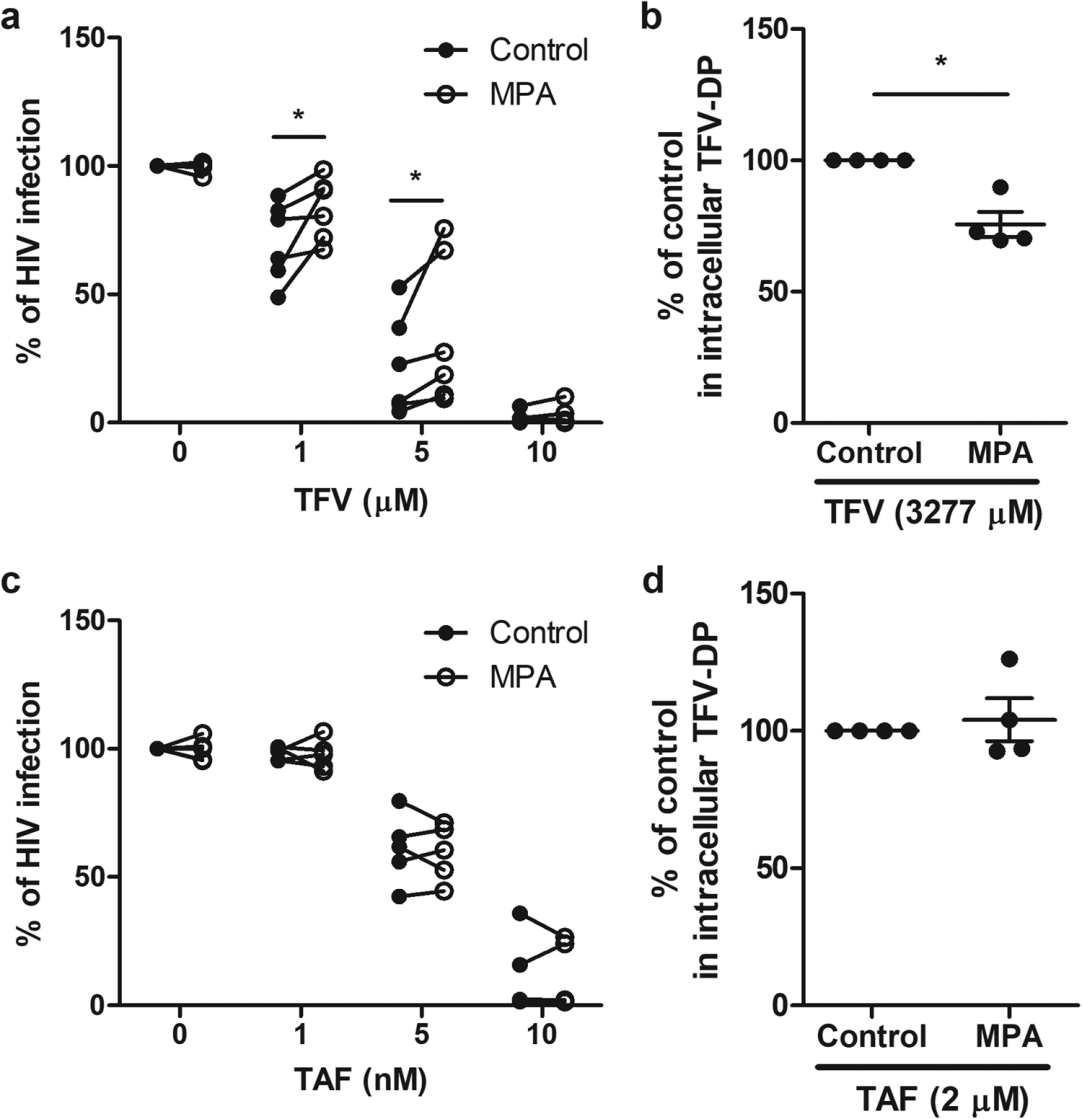
Effect of MPA on TFV and TAF inhibition of HIV infection and intracellular TFV-DP of blood CD4+ T cells. (**a** and **c**) Activated blood CD4+ T cells were pre-treated with indicated doses of TFV or TAF for 24hr and inoculated with HIV for 2hr. Following washout of virus after 2hr of culture, cells were incubated with MPA (1 × 10^−7^ M) at 5 days following infection, after which levels of p24 released in the culture media was measured by p24 enzyme-linked immunosorbent assay. Data were normalized to % of HIV infection with respect to infected controls without TFV or TAF pre-treatment and in the absence of MPA. (**a**) Percent of HIV infection on TFV treatment from 6 individual blood donors. (**c**) Percent of HIV infection on TAF treatment from 5 individual blood donors. Dark circles represent culture media in the absence of MPA and open circles represent culture media in the presence of MPA. Each circle represents one individual donor. *p < 0.05. (**b** and **d**) TFV-DP levels were measured by LC-MS/MS in purified blood CD4+ T cells pre-treated with MPA (1 × 10^−7^ M) for 24 hr followed by incubation with (**b**) TFV (3277 µM) and (**d**) TAF (2 µM) for an additional 24hr. Data were normalized to % fold change from control values. The bar represents the mean and SEM from 4 individual blood donors. Each circle represents one individual donor. *p < 0.05.

The possibility that some hormonal contraceptives interfere with TFV and/or TAF effectiveness resulting in a reduced protection against HIV acquisition prompted us to determine whether MPA might compromise the effectiveness of ARVs in preventing HIV infection of CD4+ T cells. Figure 3a shows the analyses of CD4+ T cells from 5 different blood donors, demonstrating that MPA suppresses the efficacy of TFV at 1 and 5 μM leading to increased HIV infection of CD4+ T cells. Further, it indicates that MPA alone has no effect on cell viability, since in the absence of TFV (Fig. 3 a) and TAF (Fig. 3 c) infection by HIV was not affected. An increase in secreted p24 levels indicates a significant loss of TFV protection against HIV infection in the presence of MPA compared to control TFV alone.

Given the suppression of TFV-mediated protection against HIV infection of HIV target cells by MPA, we investigated whether the effect of MPA might be due to altered intracellular concentrations of tenofovir diphosphate (TFV-DP). To determine the relationship between TFV and TFV-DP concentrations, blood CD4+ T cells were pre-treated with MPA (1 × 10^−7^M) for 24hr followed by incubation with TFV (3277 μM) for an additional 24hr prior to measuring intracellular TFV-DP. We found that the lower TFV concentrations used in the HIV studies resulted in intracellular TFV-DP concentrations that were below the limit of detection. As shown in Fig. 3b, intracellular TFV-DP in 4 separate experiments were significantly lower when cells were treated with MPA. Overall, these findings indicate that MPA reduces both protection against HIV infection by TFV as well as intracellular TFV-DP concentrations in blood CD4+ T cells.

### Lack of effect of MPA on TAF inhibition of HIV Infection of blood CD4+ T cells

Using the experimental approach described above for TFV, we asked whether MPA inhibits TAF-mediated protection of CD4+ T cells. Using concentrations of TAF identified in HIV infection dose-response experiments (Fig. 2), we selected the optimal range of TAF (1–10 nM) for preventing HIV infection. As seen in Fig. 3c in 4 separate experiments, MPA had no effect on TAF-mediated inhibition of HIV infection by HIV of blood CD4+ T cells. As a part of these studies, we asked whether MPA altered the conversion of TAF into TFV-DP. Figure 3d demonstrates that MPA had no effect on the concentration of intracellular TFV-DP when compared to control cells. These findings demonstrate, that even when evaluated at concentrations resulting in similar intracellular TFV-DP levels, MPA exerts differential effects on TFV and TAF inhibition of HIV infection of blood CD4+ T cells. Whereas MPA suppressed TFV-mediated HIV infection and lowered intracellular concentrations of TFV-DP, it had no effect on either parameter in the presence of TAF.

### Effect of MPA on TFV and TAF Inhibition of HIV Infection and Intracellular TFV-DP Concentrations in endometrial CD4+ T cells

**Figure 4:**
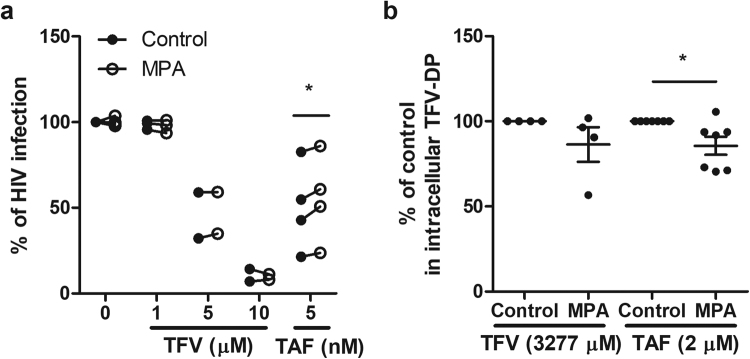
Effect of MPA on TFV and TAF inhibition of HIV infection and intracellular TFV-DP concentrations in endometrial CD4+ T cells. (**a**) Activated CD4+ T cells from endometrium (n = 4) were pre-treated with indicated concentration of TFV or TAF for 24hr and inoculated with HIV for 2hr. Levels of p24 released into the culture media presence or absence of MPA (1 × 10^−7^ M) at 5 days following infection were measured by p24 enzyme-linked immunosorbent assay. Data were normalized to % of HIV infection with respect to infected control without TFV or TAF pre-treatment and in the absence of MPA. Dark circles represent culture media in the absence of MPA and open circle represent culture media in the presence of MPA. Each circle represents one individual patient. *p < 0.05. (**b**) TFV-DP concentrations were measured by LC-MS/MS in purified CD4+ T cells isolated from endometrium pre-treated with MPA (1 × 10^−7^ M) for 24 h following treated with TFV (3277 µM) and TAF (2 µM) for 24 h. Data were normalized to % fold change from control values. Each circle represents a different patient. The mean and SEM are shown. *p < 0.05.

Recognizing that CD4+ T cells in the human FRT are unique and distinct from those in the blood^15,35^, we asked whether MPA alters TFV- and/or TAF-mediated HIV protection and intracellular TFV-DP concentrations in FRT CD4+ T cells. As seen in Fig. 4a, MPA had no effect on TFV (1, 5, and 10 μM) inhibition of HIV infection of endometrial CD4+ T cells. In contrast, MPA treatment decreased the protective effect of TAF at 5 nM against HIV infection of endometrial CD4+ T cells from 4 patients. Analysis of intracellular TFV-DP concentrations in endometrial CD4+ T cells indicated that, whereas MPA had no effect on TFV-DP concentrations derived from TFV (3277 mM), MPA significantly lowered intracellular TFV-DP concentrations derived from TAF treatment of endometrial CD4+ T cells obtained from 7 different patients (Fig. 4b). These findings indicate that MPA selectively impacts endometrial CD4+ T cells in a way that is opposite to that seen with blood CD4+ T cells both in terms of TFV and TAF protection against HIV infection and intracellular TFV-DP concentrations.

### Lack of effect of progestins and progesterone on TFV and TAF inhibition of HIV Infection and intracellular TFV-DP Concentrations in Blood CD4+ T cells

**Figure 5:**
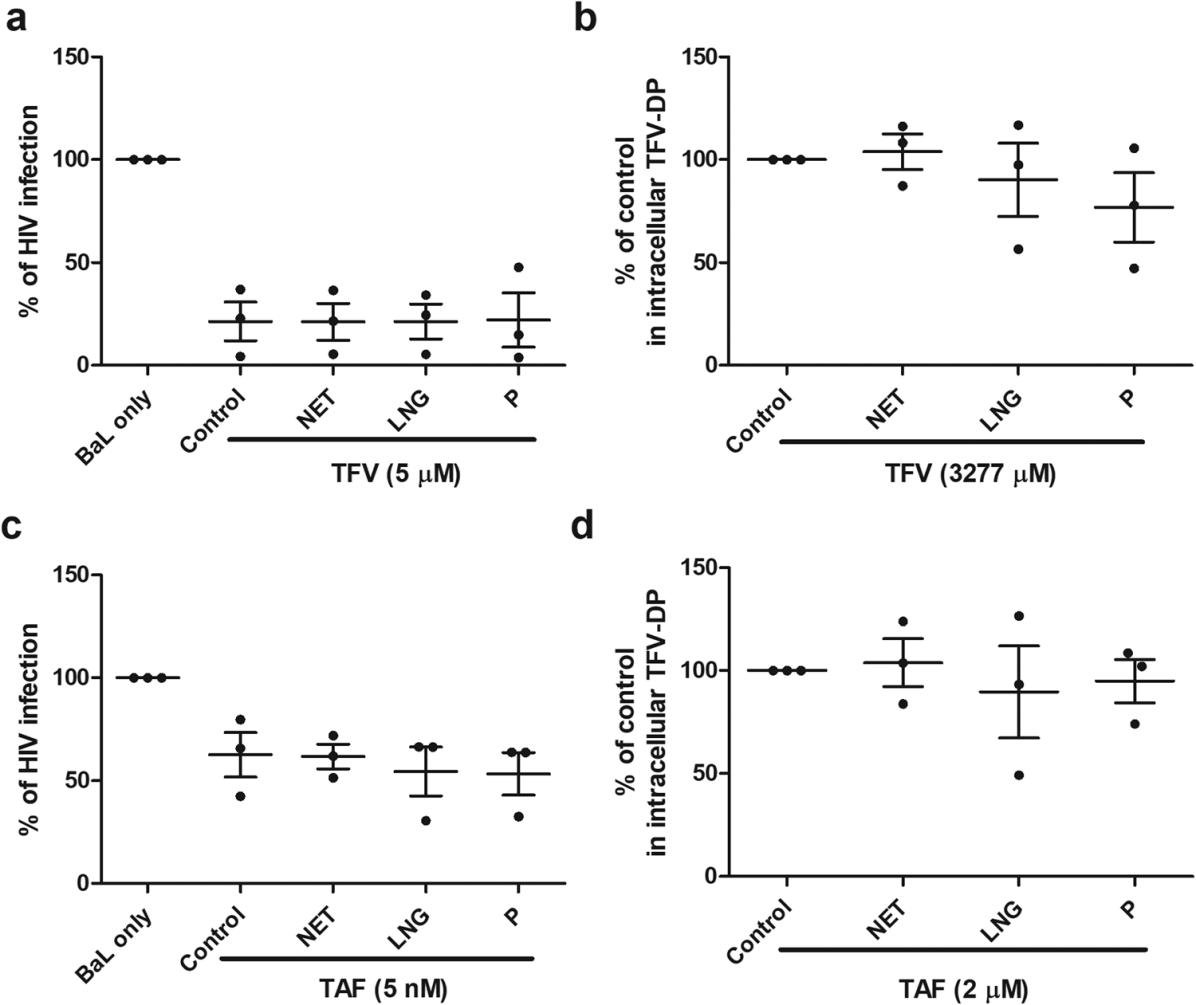
Lack of NET, LNG and progesterone on TFV and TAF inhibition of HIV infection of and intracellular TFV-DP concentration in blood CD4+ T cells. Activated blood CD4+ T cells were pre-treated with TFV at 5 µM (**a**) or TAF at 5 nM (**c**) for 24 h and inoculated with HIV for 2hr. Levels of p24 released into the culture media presence or absence of NET, LNG or progesterone (P) at 1 × 10^−7^ M for 5 days following infection were measured by p24 enzyme-linked immunosorbent assay. Data were normalized to % of HIV infection with respect to infected control without TFV or TAF pre-treated and in the absence of MPA (BaL only). TFV-DP concentrations were measured by LC-MS/MS in purified blood CD4+ T cells pre-treated with NET, LNG and progesterone at 1 × 10^−7^ M for 24hr following treated with (**b**) TFV (3277 µM) and (**d**) TAF (2 µM) for 24 hr. Data were normalized to % fold change from control values. (Each horizontal line represents the mean and SEM from 3 individual blood donors.

To determine whether the effect of MPA on TFV inhibition of blood CD4+ T cell HIV infection is a characteristic of other progestin contraceptives and naturally occurring progesterone, we examined the effects of LNG, NET and progesterone on reversing HIV infection of blood CD4+ T cells. Analysis of each progestin was undertaken under conditions identical to that used to analyze the effect of MPA on suppressing CD4+ T cell protection. The concentration of steroids used in these studies (1 × 10^−7^ M), which corresponds to or exceeds that reached in blood, was selected in order to observe any potential inhibitory effects^36^. As shown in Fig. 5a and c, we found that LNG, NET, and progesterone had no effect on TFV- or TAF-mediated protection against HIV infection of blood CD4+ T cells. Moreover, when intracellular TFV-DP concentrations derived from TFV or TAF treatment were measured (Fig. 5b and d), LNG, NET and progesterone had no effect on TFV-DP concentrations from either ARV. These findings suggest that the effects of MPA are not characteristic of all progestin compounds or of naturally occurring progesterone.

### Effect of MPA, LNG, NET and progesterone on intracellular TFV-DP from TAF treatment of endometrial CD4+ T cells

**Figure 6:**
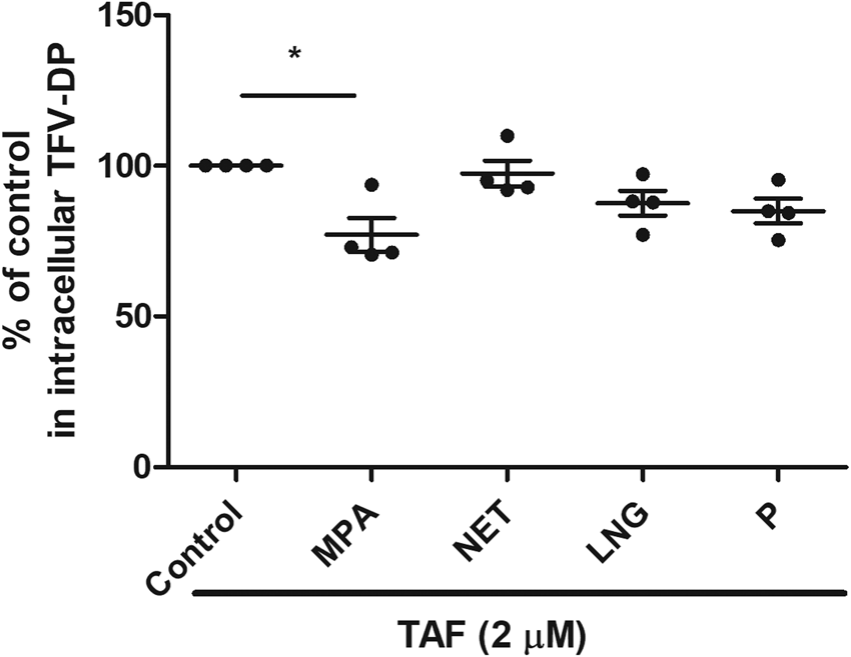
Lack of effect of NET, LNG and progesterone on intracellular TFV-DP concentrations in endometrial CD4+ T cells. TFV-DP concentrations were measured by LC-MS/MS in purified CD4+ T cells isolated from endometrium (n = 4) pre-treated with MPA, NET, LNG and progesterone (P) at 1 × 10^−7^ M for 24hr following treated with TAF (2 µM) for 24hr. Data were normalized to % fold change from control values. (Each horizontal line represents the mean and SEM from 4 women.

Since we observed a suppressive effect of MPA effect on TAF conversion to TFV-DP in endometrial CD4+ T cells (Fig. 4b), we investigated whether this occurred with other progestins. Endometrial CD4+ T cells were pre-incubated with NET, LNG, or progesterone for 24hr prior to incubation with TAF and hormonal contraceptives for an additional 24hr. As seen in Fig. 6, LNG, NET and progesterone had no effect on intracellular TFV-DP concentrations in endometrial CD4+ T cells in 4 separate experiments, in contrast to the decreased concentrations observed after MPA treatment.

## Discussion

The present study evaluated the effect of progestin contraceptives on TFV and TAF inhibition of blood and endometrial CD4+ T cell infection by HIV, and their conversion into intracellular TFV-DP. We found under conditions in which intracellular concentrations of TFV-DP are the same in blood and endometrial CD4+ T cells, that MPA but not LNG, NET or progesterone, both suppresses the protective effect of TFV on HIV infection and lowers intracellular concentrations of TDV-DP in blood CD4+ T cells. In contrast, MPA suppresses TAF inhibition of HIV infection and lowers intracellular TFV-DP concentrations in endometrial CD4+ T cells. Our *in vitro* findings demonstrate that protection against HIV infection varies with each ARV, each hormonal contraceptive, and the source of CD4+ T cells. To the best of our knowledge, these findings are the first to demonstrate that TFV and TAF anti-HIV effects can be reversed by some progestational contraceptives. Based on these findings, future HIV prevention and MPT studies should consider potential ARV/contraceptive interactions that may compromise ARV efficacy.

An important finding in our study is that TFV and TAF effectively protected blood and tissue CD4+ T cells from HIV infection *in vitro*. We also demonstrated that CD4+ T cells from the FRT (EM, CX and ECX) efficiently convert TAF into TFV-DP, with equal intracellular concentrations attained when cells were incubated with approximately 1500-fold less TAF than TFV. Interestingly, recent studies investigating tissue TAF concentrations after single dose oral administration, could not consistently detect TAF or TFV-DP in genital tissues (vagina)^30^. We have previously reported that primary epithelial cells from EM, CX and ECX convert TAF into TFV-DP^20^, with no pro-inflammatory effects, in contrast to TFV^20,37^. As TAF is being considered for PrEP after success in non-human primate studies^29^, our results suggest that once present in the FRT, TAF would be very effective in preventing HIV infection, arguing towards exploring local administration of TAF in the FRT.

A major finding in our study is that MPA treatment, but not LNG or NET, was able to decrease intracellular TFV-DP concentrations, offering a potential explanation for the loss of anti-HIV activity. The mechanisms through which MPA exerts its differential effects on CD4+ T cells from the reproductive tract are unknown but could involve cell specific actions including ARV uptake/transport and/or enzymes that convert TAF and TFV to their active forms or increase their degradation. Whereas previous studies postulated that entry of TFV and TAF into immune and non-immune cells is by diffusion or taken up by organic anionic transporters^38,39^, a recent study provides evidence that TFV is taken up by CD4+ T cells through a relatively inefficient, energy-dependent, non-receptor-mediated endocytic-like pathway^40^. In contrast, recent studies with TAF suggest that uptake into cells is by passive diffusion^41^ (Herold, B. personal communication). Beyond the level of uptake, achieving an effective concentration is the result of dosage and a composite of factors involving metabolic activation and/or degradation. The first phosphorylation of TFV to TFV-MP is known to utilize adenylate kinase (AK). The second requires a kinase with nucleotide diphosphate kinase activity. More recently, it has been shown that pyruvate kinase (PK) has a major role in the conversion of TFV-MP to TFV-DP^42^. In contrast, TAF is converted to TFV by a series of reactions initiated by Cathepsin A which results in conversion to TFV after which it utilizes the pathways described above. Our findings that MPA decreases the concentrations of TFV-derived TFV-DP in CD4+ T cells from blood and TAF-derived TFV-DP in FRT CD4+ T cells, both of which correlate with reduced anti-HIV activity, raises the likelihood that MPA exerts its effects on blood and reproductive tract CD4+ T cells through different mechanisms. Given that blood samples used in this study were different from the FRT tissues, future studies are needed to confirm these findings using matched samples, to determine the exact mechanisms involved.

The unique effect of MPA on immune cell function, in contrast to NET and LNG, has been reported by Huijbregts *et al*.^43^. One reason for the specific effects of MPA could be due to the dynamics of steroid hormone-receptor interactions. Despite sharing structural similarities, the four progestins each have different affinities for multiple steroid hormone receptors^44^. In particular, MPA has a stronger affinity for both the glucocorticoid receptor (GR) and androgen receptor (AR) than the other three progestins^45,46^. Recognizing that GR signaling is often immune-suppressive, and CD4+ T cells do not normally express nuclear progesterone receptor (PR) in non-pregnant women^47^, it is likely that MPA mediates its effects on CD4+ T cells via the GR^48^. However, T cells also express membrane PR^49^ and nuclear AR^50^. Thus, we cannot definitively rule out the contribution of these receptors to the differential effects seen in this study. Overall, these studies suggest that differential receptor expression and activity between blood and endometrial CD4+ T cells might be a mechanism by which MPA exerts cell-specific effects on TFV and TAF efficacy. Further studies are needed to define the precise hormone receptors through which progestins exert their effects in CD4+ T cells.

We found that MPA in the presence of TFV and TAF selectively increases the infection of blood and FRT CD4+ T cells by HIV respectively, whereas MPA in the absence of ARVs had no effect. Other studies, however, provide contradictory information. Sampah *et al*. showed that MPA decreased HIV infection of blood CD4+ T cells, and that this was not associated with decreased levels of activation markers (CD25, CD69, CD38) or the (co)-receptors CD4, CCR5, or CXCR4^51^. In contrast, Huijbregts *et al*. demonstrated that MPA increased the infection of blood CD4+ T cells, and prevented the downregulation of CXCR4 and CCR5 on activated CD4+ T cells^43^. The reasons for this are unclear, but are likely due to differences in experimental protocols. We introduced MPA after exposure of the CD4+ T cells to HIV, to ensure that differential activation of CD4+ T cells was not a confounding factor, whereas Sampah and Huijbregts treated CD4+ T cells with MPA prior to infection with HIV. We and Huijbregts used replication-competent HIV-BaL while Sampah employed a single-cycle pseudovirus. Further, our maximal concentration of MPA was 100 nM, while the other studies ranged between 7.5 nM (Sampah) and 1000 nM (Huijbregts). Thus, the direct effect of MPA on infection of CD4+ T cells remains unclear, but is likely affected by multiple factors including dose, time of exposure, cell activation state, and viral strain amongst others.

The mechanism involved in the selective MPA inhibition of TFV in blood CD4+ T cells but not in FRT CD4+ T cells and the inhibition of TAF in FRT CD4+ T cells but not in blood CD4+ T cells is currently under investigation. This appears to be yet another example^35^ that blood CD4+ T cells are functionally different from FRT CD4+ T cells. This appears to be yet another example^35^ that blood CD4+ T Cells are functionally different from FRT CD4+ T Cells. These results suggest that MPA differently affects either the transporters or enzymes involved in TFV/TAF metabolism in the two different cell types. Although specific effects of MPA in the two cell types on the common enzymes utilized by TFV and TFV generated from TAF to form TFV-DP cannot be ruled out, it is quite possible that the effect of MPA might be at specific steps not shared by the two drugs. Specific steps involved might include the energy requiring transport of TFV into the cell and/or the conversion of TAF via Cathepsin A (and subsequent steps) to TFV^27,40^. Studies are under way to identify the mechanisms through which MPA exerts its differential effects on blood and tissue CD4+ T cells.

Our findings may represent a potential contributing factor to the failure of HIV prevention trials with TFV, in which women were taking hormonal contraceptives, mainly MPA^7,8^. Our results suggest that under clinical conditions, when TFV concentrations are high, MPA is not sufficient to reverse anti-HIV activity. However, when TFV concentrations are low, as could be envisioned by poor adherence^11^, MPA could potentially increase the risk of HIV infection by decreasing the anti-HIV activity of TFV. No formal comparison between chemical contraceptives and HIV acquisition was performed in the majority of PrEP trials, as most of the participants were taking MPA^7,9^. However, in a secondary analysis of Partners PrEP, HIV-1 prevention among women using DMPA was efficacious and not significantly different from women using no hormonal contraception^52^. Recognizing that ARV protection was effective in this trial, these findings are consistent with our results demonstrating that high doses of ARV are effective in preventing infection in the presence of MPA. In addition, when considering topical administration of ARVs, it is unclear whether TFV reaches the upper FRT at concentrations to confer protection^17,18^, and whether this would also be true for TAF. As HIV target cells exist throughout the FRT^21–23,35,53^, MPA could increase the risk of HIV infection in the upper relative to the lower FRT, where ARV concentrations would be expected to be lower after topical (genital) administration. Less clear would be the effect of MPA when ARVs are given orally, recognizing that drug concentrations are sometimes higher in the upper FRT than the lower tract^54^.

It is worth noticing that the TFV and TAF concentrations that protect CD4+ T cells from HIV infection in our *in vitro* study are significantly lower than those measured in tissues from clinical trials^55^, which suggest that the limit for protection is much higher *in vivo* than that measured *in vitro*. Based on our previous studies^15^, it is clear that TFV and its active metabolite TFV-DP are not equally distributed between different cell types in FRT tissues. Rather, because of the concentration of TFV-DP in epithelial cells and fibroblasts, which can be 100–1000 fold higher than that measured in CD4+ T cells^15^, whether under *in vivo* conditions, relatively small changes in TFV-DP due to MPA result in increased risk of HIV infection remains to be determined.

In conclusion, our results demonstrate that MPA, but not LNG or NET, is able to reverse TFV and TAF anti-HIV protection in blood and tissue CD4+ T cells differentially. As ARVs become increasingly widespread (topical and oral), either due to incorporation in MPTs or treatment for HIV+ individuals, it is essential to understand how they interact with common contraceptives used by millions of women worldwide. Our study demonstrates another mechanism by which MPA may increase the likelihood of HIV acquisition in women – by compromising ARV efficacy. Based on these findings, future MPT studies need to consider the likelihood of ARV/contraceptive interactions that may compromise ARV efficacy, and increase the risk of HIV infection. Further, our findings highlight the need for testing ARV and hormonal contraceptive interactions in genital tissues to select the appropriate pairs that maximize anti-HIV protection in the FRT and prevent of unintended pregnancy.

## Materials and Methods

### Ethics statement

All human subject work was carried out with the approval of the Dartmouth College Institutional Review Board. Approval to use tissues was previously obtained from the Committee for the Protection of Human Subjects (CPHS), and with written informed consent obtained from the patient before surgery. All samples were anonymized, and all investigations were conducted according to the principles expressed in the Declaration of Helsinki.

### Source of tissue and blood

Human FRT tissues were obtained immediately following surgery from women who had undergone hysterectomies at Dartmouth-Hitchcock Medical Center (Lebanon, NH). Tissues from the endometrium (EM), endocervix (CX) and ectocervix (ECX) were collected from patients with benign conditions such as fibroids and prolapse (age from 27 to 51). Tissue samples were distal from the sites of pathology and were without pathological lesions as determined by a pathologist. Blood donors were anonymous, no information regarding age or hormonal status was available and only female donors were used in this study.

### Preparation of blood CD4^+^ T cells

Blood Leuko Paks from women were obtained from our IRB-approved collection facility at Dartmouth-Hitchcock Medical Center. CD4+ T cells were purified with the CD4+ T cell isolation kit (Miltenyi Biotech) following isolation of peripheral blood mononuclear cells (PBMC) by standard Ficoll density gradient centrifugation^35,56^. Freshly isolated blood CD4+ T cells were activated *in vitro* using Xvivo 15 media with Phenol Red plus Phytohemagglutinin (PHA, 2.5 µg/ml; Sigma, St Louis, MO) and IL-2 (50 U/ml, AIDS Research and Reference Reagent Program, Division of AIDS, NIAID, NIH: Human rIL-2 from Dr. Maurice Gately, Hoffmann- La Roche Inc.) for 24hr as described previously^56^. Activated CD4+ T cells were plated at a density of 1 × 10^5^ cells per well in round bottom ultra-low attachment 96-well culture plates (Corning, Corning, NY) in 0.2 ml of Immune Cell Media consisting of X-VIVO 15 Media (Lonza, Walkersville, MD) supplemented with 10% charcoal stripped human AB serum (Valley Biomedical, Winchester, VA) prior to treatment.

### Tissue processing

Tissues were rinsed with HBSS (Hanks balanced salt solution) supplemented with phenol red, 100 U/ml penicillin, 100 µg/ml streptomycin (all Thermo Scientific Hyclone, Logan, UT), and 0.35 mg/ml NaCO_3_ (Thermo Fisher Scientific, Pittsburgh, PA). Tissues were minced into 1–2 mm fragments and digested at 37 °C for 1hr using a mixture containing (final concentrations): 0.05% collagenase type IV (Sigma-Aldrich, St. Louis, MO) and 0.01% DNase (Worthington Biochemical, Lakewood, NJ) in HBSS (Invitrogen Life Technologies, Grand Island, NY). Type IV collagenase was selected based on preliminary studies to ensure non-cleavage of surface markers^35^. After digestion, cells were dispersed through a 250-µm nylon mesh screen (Small Parts, Miami Lakes, FL), washed, and resuspended in complete media consisting of DMEM/F12 medium without phenol red, supplemented with 10 mM HEPES (both GIBCO, Life Technologies, Grand Island, NY), 100 µg/ml primocin (InvivoGen, San Diego, CA), 2 mM L-glutamine, 2.5% heat-inactivated defined fetal Bovine Serum (FBS) (both from Thermo Scientific Hyclone) and 2.5% NuSerum (BD Biosciences, Bedford, MA). Epithelial cell sheets were removed from stromal cells by filtration through a 20-µm mesh filter (Small Parts). Stromal cells were then washed and counted and dead cells removed using a Dead cell removal kit (Miltenyi Biotec, Auburn, CA).

### Isolation of tissue CD4+ T cells

Following removal of dead cells, CD4+ T cells were isolated by negative magnetic bead selection with the CD4+ T cell isolation kit (Miltenyi Biotec) following instructions with minor modifications, as previously described^35^. Anti-fibroblast microbeads (Miltenyi Biotec) were added in combination with the microbeads supplied with the kit to ensure depletion of stromal fibroblasts present in the mixed cell suspension. After two rounds of negative selection, purity of the CD4+ T cell population was higher than 90%. Isolated CD4+ T cells were activated for 24 hr with PHA and IL-2 as described for blood CD4+ T cells. Activated CD4+ T cells were plated at a density of 1 × 10^5^ cells per well in round-bottom ultra-low attachment 96-well culture plates (Corning, Corning, NY) in 0.2 ml of Immune Cell Media.

### TFV and TAF preparation

TFV in powder form was obtained from AIDS Research and Reference Reagent Program (NIH AIDS Reagent Program, Division of AIDS, NIAID, NIH: Tenofovir, catalog number 10199). A stock concentration of TFV 5 mg/ml was prepared by adding 1 ml of PBS to 5 mg of TFV powder, before being diluted in stripped media to the appropriate working concentration^15,20,37^. TAF was kindly supplied by Gilead Sciences Inc. (Foster City, CA) and was dissolved in PBS at 10 mM, sterilely filtered (0.2um) and the concentration checked by absorbance using a molar extinction coefficient of 11690 at 260 nm. Subsequent dilutions of TFV and TAF were made in immune cell media to the appropriate working concentrations. TFV or TAF were added to CD4+ T cells for 24 hr prior to HIV infection or TFV-DP determination. Untreated control cells were donor-matched to treated cells. Cell viability was tested after treatment using the CellTiter 96 AQ_ueous_ One Solution cell proliferation assay (Promega, Madison, WI, USA) and trypan blue staining (HyClone Laboratories, Inc., Logan, UT) as described before^20,37^ and no changes in viability were found.

### Hormone preparation

Medroxyprogesterone 17-acetate (MPA) (Sigma-Aldrich, St. Louis, MO), Levonorgestrel (LNG) and Norethisterone (NET) and progesterone (Calbiochem, Gibbstown, NJ) were dissolved in 100% ethanol for an initial concentration of 1 × 10^−3^ M, evaporated to dryness and suspended in immune cell complete media to a concentration of 1 × 10^−5^ M. Further dilutions were made to achieve a final working concentration of 1 × 10^−7^ M. As a control, an equivalent amount of ethanol without dissolved hormone was initially evaporated.

### Intracellular TFV-DP measurement

Fresh isolated CD4+ T cells from tissues and activated CD4+ T cells from blood were pre-treated with hormone or hormonal contraceptives for 24hr prior to and during incubation with TFV (3277 μM) or TAF (2 μM) for an additional 24hr. After treatment, cells were washed, harvested and lysed in 300 µl of 70% methanol, and stored immediately at −80 °C prior to TFV-DP evaluation as previously described^13^. Intracellular TFV-DP concentrations were measured by liquid chromatography with tandem mass spectrometry (LC-MS/MS) and normalized values to fmol/million cells based on the number of cells per sample^13^. All samples were assayed blind without any information provided as to cell origin and treatment.

### HIV-infection

Activated blood or endometrial CD4+ T cells were infected as previously described with minor modifications^56^. Stock for HIV-BaL (R5) was obtained through the AIDS Research and Reference Reagent Program, Division of AIDS, NIAID, NIH, from Dr. Suzanne Gartner, Dr. Mikulas Popovic and Dr. Robert Gallo^57^. Briefly, activated blood or endometrial CD4+ T cells were treated with either TFV (1, 5 and 10 μM) or TAF (1, 5 and 10 nM) for 24hr and then washed to remove extracellular TFV or TAF. After washing, cells were incubated with HIV-BaL for 2hr at an MOI of 0.1 and then washed to remove residual virus. Fresh 0.2 ml IL-2 supplemented immune cell media with or without MPA, NET, LNG or progesterone (1 × 10^7^ M) was added to each well and cells then incubated for 5 days, with half of the media from each well collected and replaced with fresh media with or without hormone on day 3. MPA was used at a concentration of 1 × 10^−7^ M in all studies based on clinical investigations demonstrating that peak serum MPA levels reach this concentration after intramuscular administration^58–60^. This experimental sequence was followed in all HIV infection studies when it was found in initial studies that 48hr preincubation of CD4+ T cells with MPA suppressed cell activation and interfered with HIV infection. Released p24 in culture media on day 5 was measured by p24 enzyme-linked immunosorbent assay (Advanced Bioscience laboratories, Rockville, MD) following the manufacturer’s recommendations.

### Statistics

Data analysis was performed using the GraphPad Prism 5.0 (GraphPad Software, San Diego, CA). A two-sided P value < 0.05 was considered statistically significant. Comparison of two groups was performed applying Mann-Whitney U test. Comparison of three or more groups was performed applying Kruskal-Wallis, followed by Dunns-post test for multiple comparison correction. Comparison of HIV infections between CD4+ T cells from blood and tissues in the absence vs presence of hormone studies were analyzed using two-way ANOVA with Bonferroni post-test for multiple comparison correction.
